# Transcriptome analysis and comparison reveal divergence between the Mediterranean and the greenhouse whiteflies

**DOI:** 10.1371/journal.pone.0237744

**Published:** 2020-08-25

**Authors:** Yu-Jun Wang, Hua-Ling Wang, Xiao-Wei Wang, Shu-Sheng Liu

**Affiliations:** Ministry of Agriculture Key Laboratory of Agricultural Entomology, Institute of Insect Sciences, Zhejiang University, Hangzhou, China; Agricultural Research Organization Volcani Center, ISRAEL

## Abstract

Both the Mediterranean (MED) species of the *Bemisia tabaci* whitefly complex and the greenhouse whitefly (*Trialeurodes vaporariorum*, TV) are important agricultural pests. The two species of whiteflies differ in many aspects such as morphology, geographical distribution, host plant range, plant virus transmission, and resistance to insecticides. However, the molecular basis underlying their differences remains largely unknown. In this study, we analyzed the genetic divergences between the transcriptomes of MED and TV. In total, 2,944 pairs of orthologous genes were identified. The average identity of amino acid sequences between the two species is 93.6%. The average nonsynonymous (Ka) and synonymous (Ks) substitution rates and the ratio of Ka/Ks of the orthologous genes are 0.0389, 2.23 and 0.0204, respectively. The low average Ka/Ks ratio indicates that orthologous genes tend to be under strong purified selection. The most divergent gene classes are related to the metabolisms of xenobiotics, cofactors, vitamins and amino acids, and this divergence may underlie the different biological characteristics between the two species of whiteflies. Genes of differential expression between the two species are enriched in carbohydrate metabolism and regulation of autophagy. These findings provide molecular clues to uncover the biological and molecular differences between the two species of whiteflies.

## Introduction

Many whiteflies (Hemiptera: Aleyrodidae) are important pests of agriculture worldwide, such as some species of the *Bemisia tabaci* whitefly complex and the greenhouse whitefly (*Trialeurodes vaporariorum* Westwood) [1, 2]. While whiteflies of the *B*. *tabaci* species complex and the greenhouse whitefly are similar in many aspects, they differ in many features such as geographic distribution, range of host plants, virus transmission, and resistance to insecticides [2–8]. Whiteflies of the *B*. *tabaci* complex distribute in tropic and subtropical regions; some species of this whitefly complex, in particular two species, tentatively named as Middle East-Asia Minor 1 (hereafter MEAM1, formally referred to as the ‘B biotype’) and Mediterranean (hereafter MED, formally referred to as the ‘Q biotype’), have invaded many regions of the world and caused serious damage to many crops such as cotton and tomato in the last 30 years [1, 9–11]. The greenhouse whitefly *T*. *vaporariorum* (hereafter TV) inhabits the temperate regions and is a major pest of fruit, vegetable and ornamental crops in protected environment [3, 12]. In some regions, MEAM1/MED and TV coexist and show interspecific competition [13, 14]. In these regions of co-existence, they often show apparently different patterns of seasonal abundance: MEAM1/MED become predominant in seasons of relatively high temperatures, while TV becomes predominant in seasons of relatively low temperatures [15]. While all the three species of whiteflies are polyphagous, MEAM1 and MED have a wider range of host plants than TV [3, 4]. In greenhouses where MEAM1/MED and TV co-exist, they differ in patterns of within-plant distribution [16].

Another major difference between MEAM1, MED, and TV lies in their capacity of viral transmission: while whiteflies of the *B*. *tabaci* species complex, including MEAM1 and MED, transmit begomoviruses that include major agents of viral diseases of important crops such as cotton, cassava, and tomato, as well as some other groups of viruses like criniviruses and ipomoviruses, TV is a major vector of criniviruses and torradoviruses but is unable to transmit begomoviruses [2, 17]. Some criniviruses, for example *Tomato chlorosis virus*, are transmitted by both TV and MED [18, 19]. MED has developed much higher levels of resistance to major classes of insecticides than TV [6, 20, 21]. For example, MED had developed up to 1900-fold resistance to imidacloprid, and 1200-fold resistance to thiamethoxam, while TV had developed only 23.8- and 20.4-fold resistance to these two insecticides [6, 7]. However, the molecular basis underneath the differences between the whiteflies of the *B*. *tabaci* species complex and TV remains largely unknown.

RNA-seq provides an efficient approach to analyze the transcriptome of an organism and also an efficient method to discover new genes of interest [22, 23]. Pairwise comparisons between MED, MEAM1, and Asia II 3 (a native species of whitefly) have been conducted at the sequence and gene expression levels, indicating that sequence divergence of gene clusters include cytochrome P450, glutathione metabolism, and oxidative phosphorylation, and highly expression divergent genes are mainly related to basic metabolism and detoxification [24–26]. So far, several RNA-seq studies have been analyzed on whiteflies in relation to host adaption [27–30], insecticides resistance [31–33] and virus transmission [34–38]. Some detoxification genes such as cytochrome 450 monooxygenases (P450s), glutathione S-transferases (GSTs) and UDP-glucosyltransferases (UGTs) were found related with both host adaption and insecticide resistance [30–32, 39, 40]. Moreover, the detoxification gene expression patterns can shape the ability of *Bemisia* species to utilize multiple plant hosts [27].

In this study, first, we reassembled the previous version of transcriptome of MED [41] using Trinity to obtain the unigenes of similar length to those of the published TV transcriptome [42]; next, we compared the orthologous genes derived from transcriptomes of the two species; and finally, we analyzed the differential gene expression between MED and TV. Our major purpose was to find important genes that may contribute to the divergence of the two whitefly species at both genetic and expression level.

## Materials and methods

### Reads assembly, functional annotation and coding sequence prediction for MED

The raw reads of MED were downloaded from NCBI Short Read Archive (SRA), accession number: SRX018661. Before assembly, the raw reads were preprocessed by cutting off low quality base pairs within 20 bps (reads with unknown sequences ‘N’ or average quality score less than 20) of each read in the 3’ends by costumed Perl script to ensure no loss of information from paired ends. Then the preprocessed reads were assembled using the Trinity software (trinityrnaseq-r20110519) with default parameters [43].

Sequences were annotated by searching against the NCBI nr database with a cut-off E-value of 1.0E^-5^using Blastx [44]. Gene Ontology (GO) annotation was analyzed using Blast2GO software [45]. The GO terms were retrieved from Blastx hits with an e-value threshold of 1.0E^-5^. Kyoto Encyclopedia of Genes and Genomes (KEGG) pathway annotation was performed using Blastx software against the KEGG database. The best potential coding sequences (CDS) from each of the reconstructed transcripts were predicted using the software BestORF (http://www.softberry.com/berry.phtml?topic=bestorf&group=programs&subgroup=gfind) with parameters trained with *Drosophila* ESTs. Predicted CDS that start with "ATG" start codon and end with "TAA"/"TGA"/"TAG" stop codon were assumed as complete CDS.

### Identification of orthologous genes

Transcriptome sequences of TV were obtained from InsectaCentral database (http://www.insectacentral.org/). Identification of orthologous genes was performed according to the previous descriptions [24]. Briefly, transcriptome sequences of MED and TV were reciprocally blasted to obtain pairs of sequences with best hit to each other with a minimum match length of 200 bp. Then each pair of sequences were searched against the Swiss-prot database using Blastx, and only the pairs of sequences that were unambiguously mapped to the same protein (E value of 1.0E^-5^) were retained. The 5’UTR and 3’UTR regions were designated based on predicted CDS. The sequence pairs that contain predicted CDS longer than 150 bp were defined as orthologous genes.

### Analysis of sequence divergence and estimation of substitution rates

The divergence of orthologous genes at nondegenerate (nd), four-fold degenerate (4d), CpG and non-CpG regions were calculated according to the previous description [24]. The nonsynonymous sites (Ka), synonymous sites (Ks), and the Ka/Ks ratios were calculated using the YN method with the KaKs Calculator [46].

### Analysis of differential gene expression

Stock cultures of MED whitefly (*mtCO1* GenBank accession: GQ371165) and TV were maintained on cotton *Gossypium hirsutum* (Malvaceae) cv. Zhe-Mian 1793 in a climate room of 27 ± 1°C, 14 h light:10 h darkness, and 70 ± 10% relative humidity. Several hundred female adults from MED and TV were collected for further tests, with two biological replicates for each of the species. Total RNA of each sample was isolated using SV total RNA isolation system (Promega) according to the manufacturer’s protocol, respectively. Sequencing libraries were generated using NEBNext Ultra RNA Library Prep Kit for Illumina (NEB, USA). Each library was sequenced on an Illumina Hiseq 2000/2500 platform in Novogene Bioinformatics Technology Co., Ltd. (Beijing, China). Clean reads of MED and TV were obtained from NCBI BioProject PRJNA545218 (MED: SRR9141092, SRR9141088; TV: SRR141082, SRR9141090). After removing reads containing adapter or ploy-N, RSEM [47] was used to map the processed RNA-seq reads of each sample to the orthologous region of the two whiteflies [48, 49]. Differential expression analysis between the two species was conducted using edgeR [50]. Differential expression genes were selected with thresholds based on FDR P-value 0.05 and fold change 2. Goseq [51] was used for GO and KEGG enrichment analysis. ‘BH’ method was used for adjusted p-value [52].

## Results and discussions

### Reassembly the transcriptome of MED

To improve quality of the MED transcriptome previously reported, Trinity software was used to *de novo* reassemble the sequencing. The reads were assembled into 95,441 sequences (N50 = 725bp) with the length cut off of 200bp. Sequence analysis showed that 12,050 of the Trinity assembled sequences are longer than 1000 bp and 2,761 sequences longer than 2,000 bp, compared to those obtained using the SOAP method of which 4,591 sequences are longer than 1,000 and 662 sequences longer than 2,000, indicating that the new assembled transcriptome has been substantially improved.

### Annotation of predicted proteins

For functional annotation, the Trinity assembly results were searched against the NCBI non-redundant (nr) protein database using BLASTx. A total of 27,728 sequences returned significant BLAST hits (e-value<1.0E^-5^). Of them, 9,673 sequences are annotated with GO terms (E-value <1.0E^-5^), 5,398 match in “biological process”, 8,582 in “molecular function”, and 3,080 in “cellular component”. In addition, 8,469 sequences could be assigned to 293 KEGG pathways.

### Identification of orthologous genes between MED and TV

To compare the sequence divergence between MED and TV, bidirectional best hit approach, which had been widely used to identify orthologous genes [24, 53, 54], was used to find orthologous genes between the transcriptomes of the two species of whitefly. To remove potential paralogs, these putative orthologous genes were further screened against the Swiss-prot database. Only pairs of sequences that mapped unambiguously to the same protein in Swiss-prot database with an e-value < 1.0E^-5^ were selected as orthologous genes. Totally, 4,850 pairs of orthologs were kept with an average length of 591 bp and 82.02% identity (ranging from 76.6% to 100%). The untranslated region (UTR) of each sequence pair was identified based on the predicted coding region. Among the 4,850 pairs of orthologs, 57 pairs contain 5’UTR, and 54 pairs contain 3’UTR. After removing the UTRs, the CDS of all the orthologs were obtained. The CDS sequences containing unexpected stop codon were further filtered, resulting in 2,944 pairs of orthologous CDS sequences. The average length of the orthologous genes is 555 bp with an average similarity of 81.9%, which is much lower than that between the MEAM1 and MED species of the *B*. *tabaci* species complex (mean = 99.2%). The average GC content of the orthologous CDS is 42.3%, a value slightly lower than those of MEAM1 and MED species.

### Sequence divergence between the orthologous genes

Among the 2,944 orthologous gene pairs, the overall divergence in CDS is 18.1%. In non-CpG sites, the divergence is lower (16.1%); whereas in the CpG sites, the divergence (37.2%) is 2.3 times as high as that of non-CpG sites (Table 1). Nucleotides in coding regions were further classified as non-degenerative (nd) sites (any nucleotide substitutions produce amino acid change) and four-fold degenerate (4d) sites (no changes cause amino acid replacement). From a total of 1,634.08 kb of coding region sequences, 954.77 kb are nd sites, and 223.91 kb are 4d sites. At nd sites, the overall divergence is 3.7%, whereas the overall divergence at 4d sites (56.4%) is 15.4 times of that at the nd sites (Table 1). These results indicate that the nd sites evolve under extensive functional constraints because any nucleotide substitutions at nd sites will produce amino acid changes.

**Table 1:** Sequence divergence between MED and TV transcriptomes.

	%CpG	%GC	*Loci*	% differences		Compared kb
				Mean	SE	
CDS	10.02	42.34	2944			
All				18.12	0.06	1634.08
No CpG				16.06	0.06	1470.31
CpG				37.20	0.14	163.77
nd sites*	8.6	43.89	2944			
All				3.67	0.04	954.77
No CpG				3.50	0.04	872.66
CpG				5.45	0.12	82.12
4d sites^#^	17.94	35.98	2944			
All				56.38	0.18	223.91
No CpG				50.38	0.19	183.73
CpG				85.11	0.28	40.18

*nd sites: non-degenerative sites where any nucleotide substitutions produce amino acid change.

^#^4d sites: four-fold degenerate sites where no changes cause amino acid replacement.

### Synonymous and non-synonymous sites between the orthologous genes

To identify genes undergoing purifying or positive selections, rates of nonsynonymous (Ka) and synonymous (Ks) substitutions, a measure widely used to measure the intensity and mode of selection, between MED and TV ortholog pairs were estimated [55]. Among the 2,944 pairs of CDS, both a Ka and a Ks rate could be calculated for 2,742 orthologs. The mean of Ks is 2.23 (median value = 1.98), indicating that synonymous sites had substituted more than 2 times on average. The median Ks value is higher than that of the comparison between human and chicken (1.66) [56]. The Ka/Ks ratio between MED and TV (average ratio = 0.0204) is much lower than those between the three species, i.e., Asia II 3, MEAM1 and MED of the *B*. *Tabaci* whitefly complex; the average ratios are 0.198 between Asia II 3 and MEAM1, 0.201 between Asia II 3 and MED, and 0.225 between MED and MEAM 1 [24, 25]. The Ka/Ks ratio between MED and TV is even much lower than those of rodent-human (0.170) [57], chicken-human (0.052) [56], and 12 *Drosophila* species (0.06 to 0.11) [58]. The low Ka/Ks ratio is consistent with the high 4d /nd ratio, suggesting that the orthologues genes have been under high purified selection.

### Similarity of orthologous sequences

**Fig 1:**
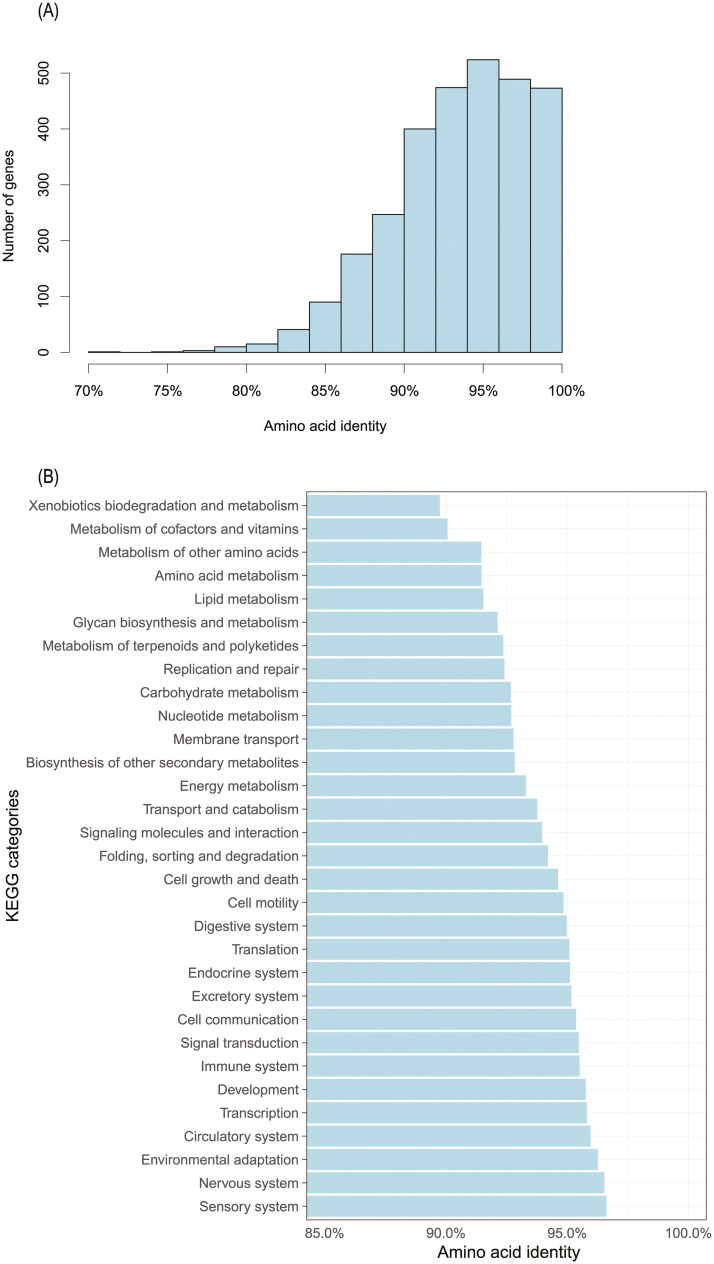
Sequence identity of orthologous. A, the distribution of amino acid identity; and B, the distribution of amino acid identity in KEGG categories.

The 2,944 pairs of orthologous CDS sequences show a mean homology of 81.9%, ranging from 70.2% to 100%. And the average homology is much lower than those shown by pairwise comparisons between species within the *Bemisia tabaci* whitefly complex (MED-MEAM1: 99.2%, MEAM1-Asia II 3: 98.3%, and MED-Asia II 3: 98.2%). Among the 2,944 orthologous gene pairs, only 18 genes show 100% homology, which is much fewer than pairwise comparisons between species within the *Bemisia tabaci* whitefly complex (MED-MEAM1: 604 and MEAM1-Asia II 3: 94) [24, 25]. This result is in line with the wider genetic distance between MED and TV compared with that between MED and MEAM1 or between MEAM1 and Asia II 3. The average identity of amino acid sequences is 93.6%, ranging from 71.7% to 100% (Fig 1a), much lower than that among species within the *Bemisia tabaci* whitefly complex (within MEAM1, MED and Asia II 3, higher than 99%). The average identity is higher than that of chicken-human (~75%) [56], rodent-human (~88%) [57], and the majority of pairwise comparisons within 7 drosophilids (*D*. *melanogaster*, *D*. *erecta*, *D*. *ananassae*, *D*. *pseudoobscura*, *D*. *mojavensis*, *D*. *virilis* and *D*. *grimshawi* except for one comparision: *D*. *melanogaster -D*. *erecta*) [58]. Among these orthologous CDS sequences, the most divergence gene pair is *Rho GTPase-activating protein 190* that is related to olfactory learning and memory in *Drosophila* [59].

Next, the orthologous genes were matched to the KEGG pathways to see the distribution of these divergent genes within each pathway (S1 Table). The most highly divergent category was xenobiotics biodegradation and metabolism, followed by categories of metabolism of cofactors, vitamins, amino acids and lipids (Fig 1b, S2 Table); these categories are also highly divergent between species of the *B*. *tabaci* whitefly complex [24, 25]. Some genes of MEAM1 and TV related to signaling pathways such as junction, spliceosome, synapse and secretion, show relatively low Ka/Ks ratio and probably have been under more purified selection (S1 Table), a pattern similar to those shown by comparison of transcriptome and genome sequences of other species of the *B*. *tabaci* whitefly complex [24, 25, 60]. This pattern of similarity might be a common feature among most species of whiteflies, and the key pathways play important roles in the divergence of different whiteflies.

### Analysis of differential expression of orthologous genes

**Fig 2:**
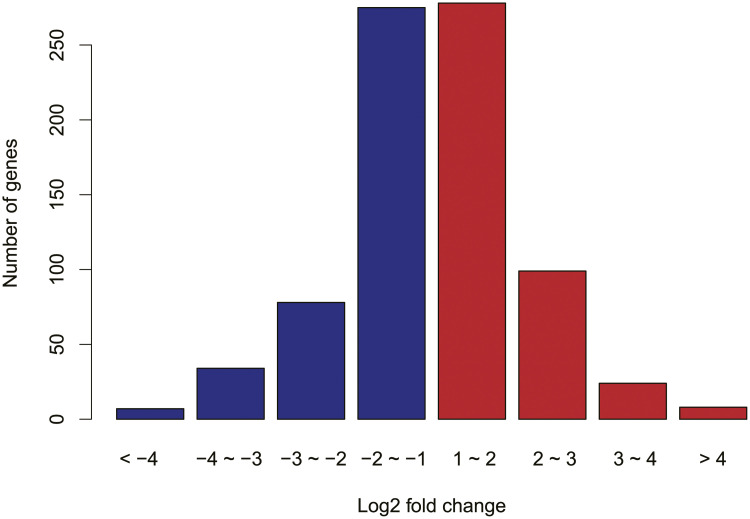
The log 2 ratio distribution of differentially expressed orthologous genes. Blue, MED over-expressed, and red, TV over-expressed.

First, RNA-seq single-end reads in each sample were mapped to *de novo* transcriptome sequences of MED or TV, respectively. The mapping rate of MED ranges from 87.5% to 89.9%, while that of TV ranges from 68.0% to 70.9% (S3 Table). Biological replication showed high reproducibility (Pearson correction was 0.98 in MED and 0.94 in TV). In order to compare differential expression in orthologous genes cross species, RNA-seq reads in each sample were mapped to orthologous regions in 2,944 pair of orthologs. Approximately 8%-10% of RNA-seq reads were mapped to the orthologous regions in each of samples (S3 Table). Genes that had absolute of log_2_ MED:TV expression ratio >1 and FDR adjusted P< 0.05 were considered as differential expression genes (DEG). In the 2,944 orthologs, 394 genes show over-expression in MED, and 409 genes show over-expression in TV (Fig 2). Some KEGG pathways show a trend to enrich (single p-value < 0.05, but adjusted p-value >0.05). Interestingly, four out of five genes of MED show over-expression in ‘regulation of autophagy’ pathway. Autophagy is a cellular degradation system, which plays an important role in homeostatic process, development, and pathology [61]. Some carbohydrate metabolism (‘galactose metabolism’, ‘pentose phosphate pathway’ and ‘fructose and mannose metabolism’) tend to be DEG enriched (Table 2; S4 Table), a feature that may be related to the strong capacity in utilizing a wide range of host plants by MED and TV. Likewise, these pathways were also DEGs enriched in the comparisons of expression divergence between MEAM1, MED and Asia II 3 [26]. However, the ‘Oxidative phosphorylation’ pathway was not DEG enriched (6 DEG out of 20, p-value = 0.39), suggesting that DEG in carbohydrate may not participate in energy metabolism but in other physiological activities. Carbohydrates not only provide energy and structural component, but also participate in various physiological activities such as protection against exposure to unfavorable temperatures, reproduction and embryonic development [62–64].

**Table 2:** Enriched KEGG pathway among the DEGs.

KEGG pathway	No. of DEGs	No. of genes	p-value	Adjusted p-value
Regulation of autophagy [PATH:ko04140]	4	5	0.016	0.941
Galactose metabolism [PATH:ko00052]	7	13	0.025	0.941
Pentose phosphate pathway [PATH:ko00030]	5	8	0.028	0.941
Fructose and mannose metabolism [PATH:ko00051]	7	14	0.034	0.941

### Genes related with amino acids, vitamins and cofactors

Phloem sap is deficient in several essential amino acids [65] and phloem sap-sucking insects harbor microbial endosymbionts to complement the requirement of these amino acids. As phloem sap-sucking insects, whiteflies harbor the primary endosymbiont *Portiera aleyrodidarum* and one to several secondary symbionts for provision of some essential amino acids, vitamins and cofactors [66–68]. For instance, in MED, seven essential amino acids (Arg, His, Lys, Val, Met, Ile and Leu) are complemented by both whitefly and symbionts, while the other three (Phe, Thr, Trp) are provided by *Portiera* alone [66]. MED and TV have high sequence divergence in categories of metabolism of cofactors, vitamins, amino acids. In order to recognize genes of the whitefly genomes rather than those of symbionts, the orthologous genes were blasted against genome sequences of *Portiera*, *Hamiltonella defensa*, MED and MEAM1. The blast results illustrate that all these genes show high identity with those of MED or MEAM1 genomes, and all top NRs hit from animals, showing that all these orthologous genes come from genomes of the whiteflies and not from the symbionts. High divergent genes are over-represented in both amino acid metabolism (26 out of 65, hyper-test p<0.05) and vitamin metabolism (21 out of 37, hyper-test p<0.05) (S5 Table). At the expression level, a few genes related to amino acids, vitamins and cofactors show significantly differential expression between MED and TV (24 out of 99) and none of the KEGG pathways are enriched by differential expression genes (S5 Table). Among these genes, some have both high sequences divergence and high expression difference such as *aminoacylase*, *biotin synthase*, and *FAD synthetase*, and may be important in amino acid and vitamin biosynthesis [60, 66] (Table 3). These divergent sequences and/or expression between MED and TV may play an important role in determining their capacity to utilize different host plants.

**Table 3:** Differential expression genes in metabolism of amino acids, vitamins and cofactors.

KO ID	KO_description	log2FC	FDR	Identity of amino acid sequences
K01939	purA, ADSS; adenylosuccinate synthase [EC:6.3.4.4]	1.24	5.92E-05	93.36%
K01580	E4.1.1.15, gadB, gadA, GAD; glutamate decarboxylase [EC:4.1.1.15]	-1.20	4.16E-04	92.52%
K14677	ACY1; aminoacylase [EC:3.5.1.14]	2.68	3.57E-06	82.71% *
K00318	PRODH; proline dehydrogenase [EC:1.5.-.-]	1.01	1.04E-04	93.22%
K00819	E2.6.1.13, rocD; ornithine—oxo-acid transaminase [EC:2.6.1.13]	-1.86	1.82E-04	95.92%
K13253	DNOS; nitric-oxide synthase, invertebrate [EC:1.14.13.39]	-1.56	3.74E-02	94.12%
K00456	CDO1; cysteine dioxygenase [EC:1.13.11.20]	-1.72	6.85E-06	90.36% *
K00108	E1.1.99.1, betA, CHDH; choline dehydrogenase [EC:1.1.99.1]	2.50	1.86E-02	92.59%
K06101	ASH1L; histone-lysine N-methyltransferase ASH1L [EC:2.1.1.43]	-2.39	1.23E-03	90.27% *
K00453	E1.13.11.11, TDO2; tryptophan 2,3-dioxygenase [EC:1.13.11.11]	4.57	3.00E-02	94.12%
K00451	HGD, hmgA; homogentisate 1,2-dioxygenase [EC:1.13.11.5]	1.60	3.57E-05	85.32% *
K09478	ACADSB; short/branched chain acyl-CoA dehydrogenase [EC:1.3.99.12]	1.83	3.01E-06	90.53% *
K01012	bioB; biotin synthase [EC:2.8.1.6]	2.99	3.98E-06	85.96% *
K01737	queD, ptpS, PTS; 6-pyruvoyltetrahydropterin/6-carboxytetrahydropterin synthase [EC:4.2.3.12 4.1.2.50]	-1.14	8.72E-04	92.22%
K03783	punA; purine-nucleoside phosphorylase [EC:2.4.2.1]	2.72	4.12E-06	95.00%
K06133	LYS5, acpT; 4’-phosphopantetheinyl transferase [EC:2.7.8.-]	1.88	1.78E-03	88.42% *
K00430	E1.11.1.7; peroxidase [EC:1.11.1.7]	-1.34	8.93E-06	93.07%
K00699	UGT; glucuronosyltransferase [EC:2.4.1.17]	1.94	1.73E-06	90.74% *
K15734	SDR16C5; all-trans-retinol dehydrogenase (NAD+) [EC:1.1.1.105]	3.41	1.18E-05	96.25%
K00953	FLAD1; FAD synthetase [EC:2.7.7.2]	-1.08	2.84E-02	78.21% *
K00861	RFK, FMN1; riboflavin kinase [EC:2.7.1.26]	-1.28	7.53E-03	86.07% *
K06125	COQ2; 4-hydroxybenzoate hexaprenyltransferase [EC:2.5.1.-]	2.53	3.49E-06	84.93% *
K06126	COQ6; ubiquinone biosynthesis monooxygenase Coq6 [EC:1.14.13.-]	1.89	3.62E-04	86.59% *
K01800	maiA, GSTZ1; maleylacetoacetate isomerase [EC:5.2.1.2]	-1.77	1.08E-03	96.97%

* Identity of amino acid sequence was lower than lower quantile of all orthologous genes.

### Genes related to metabolism of xenobiotics

The most highly divergent KEGG category is xenobiotic metabolism, which may contribute to the differences in host plant range and insecticide resistance between MED and TV. Detoxification of plant toxic compounds and resistance to insecticides can be enhanced by over-expression of GSTs, UGTs, P450s [39, 40, 69–72]. Since gene families of cytochrome P450s and UGTs are expanded in whiteflies [42, 60, 73], the number of orthologous genes in these categories are limited. Gene duplication is a mechanism of adaption to the environment [74], and the duplication of P450 genes is associated with insecticide resistance [75]. In the 2,944 orthologous genes, nine of the 13 genes related to xenobiotic metabolism show relatively high divergence of protein sequences, including one GST (79.17%), and one UGT (90.74%) (S6 Table). Four out of the seven P450 genes show relatively high divergence (Table 4), and among them *CYP4C64* was shown to be associated with imidacloprid resistance in MED [76]. At the expression level, only one GST (*GSTZ1*) is MED over-expressed, and one UGT is TV over-expressed, while all the seven P450 genes do not differ in expression between MED and TV (Table 4, S6 Table). In previous studies on the transcriptomes of MEAM1, MED and Asia II 3, numerous genes related to xenobiotic metabolism were shown to have high divergence [24, 26], while the majority of genes related to drug metabolic pathway were shown to be similarly expressed in the two invasive whiteflies MED and MEAM1 but the expression of these genes in MEAM1 and MED is higher than that in the indigenous whitefly Asia II 3 [26]. RNA-seq analysis across different species and host-plants show that the similar expression patterns of detoxification related genes associated with wide host range of whiteflies [27]. Thus, MED and TV that do not differ in expression of detoxification related genes may share a similar pattern: high detoxification gene expression, wide host range. On the other hand, a wide host range is probably associated with high insecticide resistance in whitefly [30]. Therefore, high sequence divergence and non-differential expression of detoxification related genes between MED and TV may associate with the difference in performance on plants of a wide range and insecticide resistance.

**Table 4:** Fold change of expression and identity of amino acid sequences in P450s.

Gene name of MED	Gene name of TV	Log_2_FC	FDR of DGE	Identity of protein sequences
*CYP6DB3*	*CYP6DB2*	0.63	0.71	85.07% *
*CYP4C64*	*CYP4C63*	0.30	0.34	89.94% *
*CYP301A1*	*CYP301A1*	1.98	0.10	92.93%
*CYP18A1*	*CYP18A1*	0.44	0.55	93.69%
*CYP4CR2*	*CYP4CR1*	0.64	0.08	84.52% *
*CYP301B1*	*CYP301B1*	-0.30	0.76	96.58%
*CYP380C14*	*CYP380D1*	-1.37	0.44	86.36% *

* Identity of amino acid sequence was lower than lower quantile of all orthologous genes (90.8%).

## Conclusion

In this study, we reassembled the transcriptome of MED and analyzed the divergence of sequences and expression level between MED and TV. Analysis of sequences divergence of 2,944 orthologous genes showed that these genes have been under strong purified selection. Some genes related to metabolism of xenobiotics, cofactors, vitamins, and amino acids show high protein sequence divergence between the two species of whiteflies. Genes showing differential expression were found to be enriched in carbohydrate metabolism and regulation of autophagy. These analyses provide valuable molecular references to investigate and understand the biological and molecular differences between MED and TV, and potentially differences between other species of whiteflies.

## Supporting information

S1 Table Identity of amino acid sequences and Ka/Ks in KEGG pathways. (XLSX)

S2 Table Identity of amino acid sequences and Ka/Ks in KEGG categories. (XLSX)

S3 Table Statistics of reads mapped to orthologous genes. (XLSX)

S4 Table Differential expressed genes in carbohydrate metabolism. (XLSX)

S5 Table Fold change of expression and identity of amino acid sequences in metabolism of amino acids, vitamins and cofactors. (XLSX)

S6 Table Fold change of expression and identity of amino acid sequences in metabolism of xenobiotics. (XLSX)
